# Diabetes Is an Independent Risk Factor for Severe Nocturnal Hypoxemia in Obese Patients. A Case-Control Study

**DOI:** 10.1371/journal.pone.0004692

**Published:** 2009-03-05

**Authors:** Albert Lecube, Gabriel Sampol, Patricia Lloberes, Odile Romero, Jordi Mesa, Cristina Hernández, Rafael Simó

**Affiliations:** 1 CIBER de Diabetes y Enfermedades Metabólicas Asociadas (CIBERDEM), Instituto de Salud Carlos III (ISCIII), Diabetes and Metabolism Research Unit, Institut de Recerca Hospital Universitari Vall d'Hebron, Barcelona, Spain; 2 CIBER Enfermedades Respiratorias (CIBERES), Instituto de Salud Carlos III (ISCIII), Sleep Unit, Pneumology Service, Institut de Recerca Hospital Universitari Vall d'Hebron, Barcelona, Spain; 3 Sleep Unit, Neurophysiology Service, Hospital Universitari Vall d'Hebron, Barcelona, Spain; Department of Medicine, University of Hong Kong, China

## Abstract

**Background:**

Type 2 diabetes mellitus (T2DM) and obesity have become two of the main threats to public health in the Western world. In addition, obesity is the most important determinant of the sleep apnea-hypopnea syndrome (SAHS), a condition that adversely affects glucose metabolism. However, it is unknown whether patients with diabetes have more severe SAHS than non-diabetic subjects. The aim of this cross-sectional case-control study was to evaluate whether obese patients with T2DM are more prone to severe SAHS than obese non-diabetic subjects.

**Methodology/Principal Findings:**

Thirty obese T2DM and 60 non-diabetic women closely matched by age, body mass index, waist circumference, and smoking status were recruited from the outpatient Obesity Unit of a university hospital. The exclusion criteria included chronic respiratory disease, smoking habit, neuromuscular and cerebrovascular disease, alcohol abuse, use of sedatives, and pregnancy. Examinations included a non-attended respiratory polygraphy, pulmonary function testing, and an awake arterial gasometry. Oxygen saturation measures included the percentage of time spent at saturations below 90% (CT90). A high prevalence of SAHS was found in both groups (T2DM:80%, nondiabetic:78.3%). No differences in the number of sleep apnea-hypopnea events between diabetic and non-diabetic patients were observed. However, in diabetic patients, a significantly increase in the CT90 was detected (20.2±30.2% vs. 6.8±13,5%; p = 0.027). In addition, residual volume (RV) was significantly higher in T2DM (percentage of predicted: 79.7±18.1 vs. 100.1±22.8; p<0.001). Multiple linear regression analyses showed that T2DM but not RV was independently associated with CT90.

**Conclusions/Significance:**

T2DM adversely affects breathing during sleep, becoming an independent risk factor for severe nocturnal hypoxemia in obese patients. Given that SAHS is a risk factor of cardiovascular disease, the screening for SAHS in T2DM patients seems mandatory.

## Introduction

Sleep apnea-hypopnea syndrome (SAHS) has been well established as an independent risk factor for hypertension, myocardial infarction, and stroke [1]. Obesity is the most important determinant of SAHS with more than 50% of patients having a BMI higher that 30 kg/m^2^ [2]. In recent years, increasing evidence has appeared suggesting an association between SAHS and type 2 diabetes mellitus (T2DM), two common disorders that occur with increased frequency in the obese population [3]. The available data suggest that long-term exposure to intermittent hypoxia and sleep fragmentation increases sympathetic nerve activity, contributing to disorders of glucose metabolism [4]. In this regard, a high prevalence of fasting hyperglucemia, insulin resistance, and T2DM has been found among SAHS patients in comparison with healthy subjects [5].

Alternatively, some studies propose that insulin resistance and chronic hyperglycemia might contribute to the development of SAHS. Vgontzas *et al* [6] found that women with polycystic ovary syndrome, a condition associated with insulin resistance, were much more likely than controls to have SAHS and daytime sleepiness, suggesting that insulin resistance is a mediator of SAHS in humans. Additionally, an analysis of the *Sleep Heart Health Study* found that subjects with clinically diagnosed T2DM had increased sleep-disordered breathing and more severe sleep hypoxemia, although after correcting for the main factors involved in the development of SAHS the difference in sleep hypoxemia was eliminated [7]. Finally, in non-obese rats, Ramadan *et al* [8] have recently shown direct evidence for the contribution of insulin resistance in the development of apneic episodes, and how treatment with metformin, a drug currently used to raise insulin sensitivity, reversed and prevented these episodes. However, it is unknown whether or not diabetic patients have more severe apneic episodes than non-diabetic patients.

On this basis, the aim of this case-control study was to evaluate the prevalence and characteristics of SAHS according to the presence of T2DM. For this purpose, polygraphy and respiratory function data were compared between diabetic and non-diabetic patients closely matched by the most important variables that could affect the prevalence and severity of sleep-disordered breathing.

## Materials and Methods

### Ethic statement

Informed written consent was obtained from all participants, and the human ethics committee from the Hospital Universitari Vall d'Hebrón approved the study.

### Description of patients

A total of thirty consecutive morbidly obese type 2 diabetic women attending the outpatient Obesity Unit of a university hospital were recruited for the study over a 18-months period. The main clinical features are displayed in table 1. Sixty non-diabetic women attending the same Obesity Unit served as control group. Both groups were carefully matched by age, BMI, and waist circumference.

**Table 1:** Main clinical characteristics of subjects included in the study.

	T2DM	Non T2DM	P
**n (women)**	30	60	-
**Fasting glucose (mg/dl)**	162.43±62.93	100.96±12.20	<0.001
**HbA1c (%)**	7.70±1.12	5.91±0.36	<0.001
**Total cholesterol (mg/dl)**	201.82±33.76	204.04±43	0.799
**LDL cholesterol (mg/dl)**	126.86±32.04	121.67±36.07	0.520
**HDL cholesterol (mg/dl)**	47.33±10.09	49.53±18.54	0.570
**Triglycerides (mg/dl)**	175.78±62.70	129.53±45.69	<0.001
**Systolic blood pressure (mmHg)**	138.96±21.04	138.47±21.35	0.923
**Diastolic blood pressure (mmHg)**	87.81±14.07	92.80±11.17	0.088
**Insulin treatment, n (%)**	6 (20.0)	-	-
**Oral hypoglycemic agents, n (%)**	19 (63.3)	-	-
**Microangyopathy, n (%)** *	4 (13.3)	-	-

* Non-proliferative retinopathy was present in three patients whereas microalbuminuria was present in the last one.

A complete physical examination was performed with special attention to neurological, cardiopulmonary, and ear, nose and throat evaluations. The exclusion criteria were: chronic respiratory disease, smoking habit, clinical history of neuromuscular disease, narcolepsy, stroke, transient ischemic attack, chronic heart failure, craniofacial abnormalities, abuse of alcohol or use of sedatives, pregnancy, and endocrinological diseases apart from obesity and diabetes mellitus. T2DM was defined according to the criteria recommended by the Expert Committee on the Diagnosis and Classification of Diabetes [9]. The degree of sleepiness was evaluated using the Epworth Sleepiness Scale (ESS), a widely used questionnaire based on the tendency to fall asleep during various daytime situations [10].

### Measurement of sleep disorders of breathing and respiratory function data

A previously validated non-attended respiratory polygraphy was performed at patients' homes with a Somnea polygraph (Compumedics, Abbotsford, Australia) which records nasal airflow (nasal cannula), respiratory effort (chest and abdominal bands), snoring, body position and finger pulse oxymetry [11]. After the performance of the study patients were required to answer a questionnaire on self-perception of sleep quality, subjective number of hours slept and number of awakenings. An expert scorer blinded to the study reviewed all sleep studies manually. Studies with less than 5 hours of correct signal recording were ruled out and repeated. An apnea was defined as cessation of airflow with duration of at least 10 seconds. Differentiation was made between obstructive and central apneas according to the respiratory effort channels (presence or absence of thoracoabdominal movements). Hypopnea was defined as a >50% reduction in nasal cannula tracing with a duration of at least 10 seconds associated with a cyclical dip in arterial oxygen saturation (SaO_2_) ≥3%. The apnea-hypopnea index (AHI) was defined as the sum of apneas plus hypopneas divided by time in bed. On this basis, SAHS was defined as an AHI≥10 events/hour, and patients were divided into non-SAHS (AHI<10 events/hour), mild SAHS (AHI between 10 and 20 events/hour), moderate SAHS (AHI between 21 and 29 events/hour), and severe SAHS (AHI >30 events/hour). Three oxygen saturation measures were assessed: the cumulative percentage of time spent with oxygen saturations below 90% (CT90), and the lowest and the average SaO_2_.

Forced spirometry and static pulmonary volume measurements were performed using a MasterLab apparatus (MasterLab; Jaeger; Würzburg, Germany). All tests were performed following European Respiratory Society guidelines [12]. Static pulmonary volumes were measured using the plethysmography method. The theoretical values proposed by Roca *et al* [13] were applied for spirometry, and values proposed by the European Respiratory Society for static volumes [12].

Room air arterial blood gas sampling was performed according to standard guidelines. Briefly, after patients had been sitting for at least 10 minutes samples were anaerobically drawn into preheparinized syringes following the administration of local anesthesia in the area of the radial artery. Air bubbles were removed and each sample was taken immediately for analysis using an IL 682 co-oxymeter (Instrumentation Laboratories, Lexington; MA).

### Statistical analysis

Normal distribution of the variables was evaluated using the Kolmogorov-Smirnov test. Data were expressed either as the mean (SD) or percentage. Given their skewed distribution, AHI and oxygen saturation data were expressed as median (range). For parametric tests, AHI was logarithmically transformed to achieve a normal distribution. Comparisons between groups were performed using Student *t* tests for continuous variables and the *χ* ^2^ test for categorical variables. Oxygen saturation data (the lowest and the average SaO_2_) were compared using non-parametric tests (Mann-Whitney U test). The relationship between the continuous variables was examined by the Pearson linear correlation test.

A stepwise multiple linear regression analyses to explore the variables independently related to SAHS severity was performed. CT90 was considered as the dependent variable, and the independent variables included in the analyses were: age, BMI, AHI (log), arterial oxygen pressure (PaO_2_), arterial carbon dioxide pressure (PaCO_2_), residual volume (RV, percentage of predicted), forced expiratory volume in 1 second to forced vital capacity ratio (FEV1/FVC), somnolence score, and the presence or absence of T2DM.

All *p* values were based on a two-sided test of statistical significance. Significance was accepted at the level of *p*<0.05. Statistical analyses were performed with the SSPS statistical package (SPSS Inc, Chicago, Illinois).

## Results

The main clinical features and pulmonary parameters of the study population are presented in table 2. A high prevalence of SAHS, with almost 3 out of 4 subjects affected, was found in both groups. Diabetic and non-diabetic patients showed a similar prevalence of mild (37.5% *vs.* 34.0%), moderate (20.8% *vs.* 21.3%), and severe (41.6% *vs.* 44.6%) SAHS. When polygraphic results were analysed no significant differences in the number of apnea-hypopnea events were observed between diabetic and non-diabetic patients. In addition, the lowest and the average oxygen saturation levels were also similar in both groups. When the rate (events/hour) of apneas and hypopneas were evaluated separately, we observed a similar frequency in diabetic patients than in nondiabetic patients: 2.8 (0–87.4) *vs.* 2.8 (0–50.0), p = 0.956 for apneas, and 16.1 (0.7–50.0) *vs.* 17.0 (0.7–71.4), p = 0.969 for hypopneas. However, diabetic patients spent a significantly higher percentage of time with oxygen saturation below 90% in comparison with non-diabetic subjects (20.1%±30.2 *vs.* 6.8%±13.5, p = 0.027).

**Table 2:** Main clinical features and pulmonary parameters of subjects included in the study.

	T2DM	Non T2DM	P
**n**	30	60	-
**Age (years)**	43.2±8.0	42.1±8.0	0.529
**BMI (Kg/m^2^)**	49.1±6.3	49.1±6.4	0.989
**Waist circumference (cms)**	130.8±12.2	129.8±13.4	0.746
**Polygraphy data:**			
**SAHS, n (%)**	24 (80.0)	47 (78.3)	0.855
**AHI (events/h)**	20.00 (4.90–114.0)	22.70 (0.70–78.0)	0.503
**CT90 (%)**	20.2±30.2	6.8±13.5	0.027
**Average SaO_2_ (%)**	94.0 (75.0–98.0)	94.0 (88.0–98.0)	0.277
**Lowest SaO_2_ (%)**	78.0 (49.0–90.0)	82.0 (49.0–97.0)	0.115
**Pulmonary function test:**			
**FVC (%)** *	87.3±17.1	89.9±13.7	0.447
**FEV1 (%)** *	91.2±20.2	97.3±14.3	0.114
**FEV1/FVC (%)**	81.4±9.1	85.0±5.0	0.058
**TLC (%)** *	97.2±14.0	94.3±11.6	0.369
**RV (%)** *	100.1±22.8	79.7±18.1	<0.001
**Arterial blood samples:**			
**PaO_2_ (mmHg)**	82.3±11.4	83.6±8.1	0.574
**PaCO_2_ (mmHg)**	39.7±5.2	38.9±3.9	0.441
**Epworth Sleepiness Scale**	7.3±4.6	6.5±4.1	0.453

Data are mean±SD and median (range). T2DM: type 2 diabetes mellitus; BMI: body mass index; SAHS: sleep apnea-hypopnea syndrome; AHI: apnea-hipoapnea index; CT90: percentage of time spent with oxygen saturations below 90%; SaO2: arterial oxygen saturation; FVC: forced vital capacity; FEV1: forced expiratory volume in 1 second; TLC: total lung capacity; RV: residual volume; PaO_2_: arterial oxygen pressure; PaCO_2_: arterial carbon dioxide pressure.

* Value expressed as a percentage of the predicted value.

Pulmonary function tests showed spirometric and static lung volumes mean values in the normal range and without significant differences between the two groups except a greater residual volume (RV) in diabetic patients, (100.0±22.8 *vs.* 79.7±18.1; p<0.001) (Table 2). Regarding somnolence, an ESS>10 was present in 6 (20.00%) of the obese women with T2DM and in 8 (13.33%) of the control group (p = 0.362)

The univariate analysis showed that CT90 was correlated with several parameters obtained from non-attended polygraphy and arterial blood samples including age, AHI(log), average and lowest SaO_2_, forced vital capacity (FVC), total lung capacity (TLC), PaO_2_, PaCO_2_, and the ESS (Table 3). However, we did not find any correlation between CT90 and BMI (r = 0.162, p = 0.127), RV (r = 0.097, p = 0.416) nor FEV1/FVC ratio (r = −0.176, p = 0.107).

**Table 3:** Correlations of percentage of time spent with oxygen saturations below 90% (CT90) with clinical and metabolic variables obtained in the univariate analyses.

Variable	r	p
**Age**	0.242	0.022
**BMI**	0.162	0.127
**AHI**	0.547	<0.001
**Average SaO_2_**	0.856	<0.001
**Lowest SaO_2_**	−0.632	<0.001
**FVC**	−0.388	<0.001
**FEV1/FVC ratio**	−0.176	0.107
**TLC**	−0.241	0.040
**RV**	0.097	0.416
**PaO_2_**	−0.477	<0.001
**PaCO_2_**	0.475	<0.001
**ESS**	0.302	0.005
**Fasting glucose**	0.097	0.366
**HbA1c**	0.102	0.549
**Systolic blood pressure**	0.164	0.143
**Diastolic blood pressure**	0.195	0.081
**Total cholesterol**	−0.017	0.885
**LDL cholesterol**	0.009	0.936
**HDL cholesterol**	−0.112	0.335
**Triglycerides**	0.072	0.537

AHI: apnea-hipoapnea index; SaO2: arterial oxygen saturation; FVC: forced vital capacity (percentage of predicted); TLC: total lung capacity (percentage of predicted); RV: residual volume (percentage of predicted); PaO_2_: arterial oxygen pressure; PaCO_2_: arterial carbon dioxide pressure; ESS: Epworth Somnolence Score.

Multiple linear regression analysis showed that type 2 diabetes (beta = 0.220, p = 0.007), together with PaO_2_ (beta = −0.222, p = 0.013), PaCO_2_ (beta = 0.227, p = 0.013), AHI(log) (beta = 0.387, p<0.001), and the ESS (beta = 0.184, p = 0.028), were independently associated with CT90 (R^2^ = 0.582) (table 4).

**Table 4:** Multiple linear regression analysis of variables associated with the percentage of time spent with oxygen saturations below 90% (CT90).

	Beta	p
**AHI (log)**	0.387	<0.001
**PaCO_2_ (mmHg)**	0.227	0.013
**T2DM (yes/no)**	0.220	0.007
**PaO_2_ (mmHg)**	−0.222	0.013
**BMI (Kg/m^2^)**	−0.094	0.279
**ESS**	0.184	0.028
**Age (yrs)**	−0.001	0.859
**R^2^**	**0.582**	

Beta: Standardized partial regression coefficient. AHI: apnea-hypopnea index; PaCO_2_: arterial carbon dioxide pressure; T2DM: type 2 diabetes mellitus; PaO_2_: arterial oxygen pressure; BMI: body mass index; ESS: Epworth Sleepiness Scale; RV: residual volume (percentage of predicted); FEV1/FVC: forced expiratory volume in 1 second to forced vital capacity ratio.

## Discussion

To the best of our knowledge, this is the first study to provide evidence that T2DM is an independent risk factor for severe nocturnal hypoxemia in morbidly obese patients. In fact, the cumulative percentage of time spent with oxygen saturation below 90% was three-fold higher in diabetes patients than in non-diabetic subjects matched by age, gender, BMI and waist circumference. This is an important finding because sleep related hypoxia in SAHS patients is known to be a major stimulus leading to oxidative stress and endothelial dysfunction [14], and may contribute to the increased risk of fatal and non-fatal cardiovascular events observed in patients with severe SAHS [1]. In our patients, this effect may be an additional source of oxidative stress to that associated with T2DM [15]. Therefore, due to its high prevalence and severity it seems mandatory to investigate SAHS, and in particular nocturnal hypoxemia, in this subset of diabetic subjects.

As previously described, sleep hypoxemia in our patients was related to awake blood gases and AHI [16]. The deleterious effect of the presence of T2DM on CT90 in obese SAHS patients observed in our study may be mediated through several mechanisms. It has been suggested that insulin resistance, through both the activation of nuclear factor kappa B by increased levels of cytokines (i.e., IL-6 and TNF-alpha) and the releasing of free fatty acids by excess adipose tissue, is involved in the pathogenic mechanisms leading to SAHS in humans [17]. Data supporting this hypothesis include the significant reduction of sleepiness and sleep apnea/hypopnea events in obese patients after the administration of etanercept, a TNF-alpha antagonist [18]. In addition, Ramadan *et al* [8] demonstrated that insulin resistance was one of the primary mechanism leading to sleep apnea in rats.

A deleterious effect of diabetes on central respiratory control attributed to autonomic neuropathy has also been described [19]. Resnick *et al* [7], using cross-sectional data from the Sleep Heart Health Study, after adjustment for BMI and other potential confounders, found that patients with diabetes had more episodes of periodic breathing, an abnormality of the central control of ventilation, than did those subjects without diabetes. A limitation of the study is that the presence of diabetes was based on self-report or on the use of hypoglycaemic medications. Therefore, it is possible that a substantial number of individuals were misclassified as “non-diabetic”. However, laboratory based investigations show that diabetic patients with autonomic neuropathy are more likely to have not only central, but also obstructive sleep apnea than diabetic patients without autonomic neuropathy [19]. This association is also present in non-obese diabetic patients and seems independent of the severity of their dysautonomy [20]. The underlying mechanism has to be elucidated, but an impairment of the upper airway reflexes, possibly due to alterations to the autonomic nervous fibres involved in their regulation, could lead to an inability of individuals with diabetes to respond appropriately to sleep apneas. In addition, an impairment of ventilatory drive associated to leptin resistance has been shown in obese patients [21]. These mechanisms could lead to a greater instability of the upper airway and to a more profound hypoxemia observed in our obese diabetic patients. Furthermore, it has been suggested that cardiac dysfunction (diminished heart rate variability and left ventricular dysfunction), common in diabetic neuropathy, may prolong circulatory time and cause a delay in feedback loops involving CO_2_ and O_2_ central and peripheral chemorreceptors [7]. Supporting this hypothesis, streptozotocin-induced diabetic rats exhibited a significant reduction in the ventilatory response to hypercapnic and hypoxic challenges which was prevented with insulin treatment [22]. Consequently, our results raise the central issue of whether the normalization of blood glucose levels in humans can significantly improve the severity of sleep apnea.

The results obtained in the present study, in which type 2 diabetic subjects showed a significant increase in RV accompanied with a decrease in the FEV1/FVC ratio, reinforce previous data [23]. However, it should be noted that our patients had a mean spirometric and stating lung volume values in the normal range. We feel that this fact has limited our ability to detect the effect of pulmonary function test on sleep hypoxemia detected in previous works. An impairment in pulmonary carbon monoxide diffusing capacity, which has been reported to be altered in some T2DM [24], can not be ruled out in our patients. However, we believe that the magnitude of the impairment previously described would not explain the sleep hypoxemia found in the present study. Although it is tempting to suggest a relationship between increased RV and severe nocturnal hypoxemia in T2DM patients with SAHS, no correlation between RV and data obtained from the non-attended polygraphy, including CT90, was observed. These findings strongly suggest that the higher severity of SAHS observed in T2DM was unrelated to pulmonary function tests.

There are some potential limitations that should be taken into account in evaluating the results of our study. First, this was a cross-sectional study and, therefore, a causal link between T2DM and sleep hypoxemia can not be drawn. In this regard, studies addressed to determining whether the normalization of blood glucose levels could improve SAHS events or hypoxia are warranted. Second, patients were not matched for neck circumference. However, it has been described that visceral fat is the main risk factor for SAHS in obese subjects, whereas the AHI does not correlate with subcutaneous fat in the neck or parapharingeal regions [25]. Third, we have considered a selected population of morbidly obese women and, consequently, further studies in non-obese T2D patients are required.

In conclusion, T2DM adversely affects breathing abnormalities during sleep in morbid obese patients. Given that SAHS is a risk factor of cardiovascular disease, the screening for SAHS in these patients seems mandatory. Further studies to define not only the mechanisms through which T2DM promotes severe hypoxemia in SAHS but also to determine whether or not blood glucose control could arrest or prevent this deleterious effect are needed.

## References

[1] Marin JM, Carrizo SJ, Vicente E, Agustí AG (2005). Long-term cardiovascular outcomes in men with obstructive sleep apnoea-hypopnoea with or without treatment with continuous positive airway pressure: an observational study.. Lancet.

[2] Young T, Peppard PE, Taheri S (2005). Excess weight and sleep-disordered breathing.. J Appl Physiol.

[3] Tasali E, Mokhlesi B, Van Cauter E (2008). Obstructive sleep apnea and type 2 diabetes. Interacting epidemics.. Chest.

[4] Peltier AC, Consens FB, Sheikh K, Wang L, Song Y (2007). Autonomic dysfunction in obstructive sleep apnea is associated with impaired glucose regulation.. Sleep Med.

[5] Punjabi NM, Polotsky VY (2005). Disorders of glucose metabolism in sleep apnea.. J Appl Physiol.

[6] Vgontzas A, Legro R, Bixler E, Grayev A, Kales A (2001). Polycystic ovary syndrome is associated with obstructive sleep apnea and daytime sleepiness: role of insulin resistance.. J Clin Endocrinol Metab.

[7] Resnick HE, Redline S, Shahar E, Gilpin A, Newman A (2003). Sleep Heart Health Study: Diabetes and sleep disturbances: findings from the Sleep Heart health Study.. Diabetes Care.

[8] Ramadan W, Dewasmes G, Petitjean M, Wiernsperger N, Delanaud S (2007). Sleep apnea is induced by a high-fat diet and reversed and prevented by metformin in non-obese rats.. Obesity (Silver Spring).

[9] American Diabetes Association (2005). Diagnosis and Classification of Diabetes Mellitus.. Diabetes Care.

[10] Johns MW (1991). A new method for measuring daytime sleepiness: the Epworth sleepiness scale.. Sleep.

[11] Lloberes P, Sampol G, Levy G, Aristizabal D, Sagales T (2001). Influence of setting on unattended respiratory monitoring in the sleep apnoea-hypopnoea syndrome.. Eur Respir J.

[12] Quanjer PH, Tammeling GJ, Cotes LE, Pedersen OF, Peslin R (1996). Lung volumes and forced ventilatory flows.. Eur Respir J.

[13] Roca J, Sanchis J, Agusti-Vidal A, Segarra F, Navajas D (1986). Spirometric reference values from a Mediterranean population.. Bull Eur Physiopathol Respir.

[14] Lavie L (2003). Obstructive sleep apnoea syndrome. An oxidative stress disorder.. Sleep Med Rev.

[15] Hartge MM, Unger T, Kintscher U (2007). The endothelium and vascular inflammation in diabetes.. Diab Vasc Dis Res.

[16] Bradley TD, Martinez D, Rutherford R, Lue F, Grossman RF (1985). Physiological determinants of nocturnal arterial oxygenation in patients with obstructive sleep apnea.. J Appl Physiol.

[17] Williams S, Scharf SM (2007). Obstructive sleep apnea, cardiovascular disease, and inflammation-is NF-kappa B the key?. Sleep Breath.

[18] Vgontzas AN, Zoumakis E, Lin HM, Bixler EO, Trakada G (2004). Marked decrease in sleepiness in patients with sleep apnea by etanercept, a tumor necrosis factor-α antagonist.. J Clin Endocrinol Metab.

[19] Ficker JH, Dertinger SH, Siegfried W, König HJ, Pentz M (1998). Obstructive sleep apnoea and diabetes mellitus: the role of cardiovascular autonomic neuropathy.. Eur Respir J.

[20] Bottini P, Dottorini ML, Cristina Cordomi M, Casucci G, Tantucci C (2003). Sleep-disordered breathing in nonobese diabetic subjects with autonomic neuropathy.. Eur Respir J.

[21] Campo A, Frühbeck G, Zulueta JJ, Iriarte J, Seijo LM (2007). Hyperleptinemia, respiratory drive and hypercapnia response in obese patients.. Eur Respir J.

[22] Hein MS, Schlenker EH, Patel KP (1994). Altered control of ventilation in streptozotocin-induced diabetic rats.. Proc Soc Exp Biol Med.

[23] Davis WA, Knuiman M, Kendall P, Grange V, Davis TM (2004). Fremantle Diabetes Study: Glycemic exposure is associated with reduced pulmonary function in type 2 diabetes: the Fremantle Study.. Diabetes Care.

[24] Guazzi M, Oreglia I, Guazzi MD (2002). Insulin improves alveolar-capillary membrane gas conductance in type 2 diabetes.. Diabetes Care.

[25] Shinohara E, Kihara S, Yamashita S, Yamane M, Nishida M (1997). Visceral fat accumulation as an important risk factor for obstructive sleep apnoea syndrome in obese subjects.. J Intern Med.

